# Book Reviews

**Published:** 1906-12

**Authors:** 


### BOOK REVIEWS.

---

SURGERY: ITS PRINCIPLES AND PRACTICE. In five volumes. By 66 eminent surgeons. Edited by W. W. Keen, M.D., LL.D., Hon. F.R.C.S., England and Edinburgh, Professor of the Principles of Surgery and of Clinical Surgery, Jefferson Medical College, Philadelphia. Vol. I. Octavo of 983 pages, with 261 text illustrations and 17 colored plates. Philadelphia and London: W. B. Saunders Company, 1906. Per volume, Cloth, $7.00 net; Half Morocco, $8.00 net.

The first volume of this magnificent work on surgery is devoted to the following subjects: "Narrative of Surgery," containing brief biographies of celebrated foreign and American surgeons, by J. G. Mumford; "Surgical Pathology," by G. W. Crile; "Examination of the Blood," by J. C. DaCosta; "Infection and Immunity," by C. Hektoen; "Inflammation," by J. G. Adami; "Suppuration, Abscess and Fistula," by L. Freeman; "Mortification and Gangrene," by the same author; "Ulceration and Ulcers," by the same; "Process of Repair," by F. C. Wood; "Thrombosis and Embolism," by C. H. Frazier; "Erysipelas," by the same; "Tetanus, Diseases Caused by Special Infections, Diseases Derived Directly from Animals and Reptiles," by Frazier; "The Traumatic Fevers," by E. A. Smith; "Scurvy," by Frazier; "Rickets," by Nichols; "Surgical Tuberculosis," by DaCosta; "Chanceroid and Syphillis," by E. Martin; "Tumors," by Bland-Sutton; "Wounds and Contusions," by Crile. The volume is handsomely bound and excellently printed. There are a large number of illustrations and the volume is carefully and fully indexed.

---

DISEASES OF THE DIGESTIVE SYSTEM. Edited by Frank Billings, M. D., Professor Medicine, University of Chicago; Professor of Medicine and Dean of Faculty, Rush Medical College, Chicago, Ill.

This is an authorized translation from Die Deutsche Klinik and the third volume in Modern Clinical Medicine—the first volume, "Infectious Diseases," edited by J. C. Wilson, A. M., M. D., Philadelphia; the second volume, "Diseases of Metabolism and of the Blood, Animal Parasites, Toxicology," edited by Richard C. Cabot, M.D.

This third volume should receive a very warm reception, for to-day "Diseases of the Digestive Tract" stand in the forefront of subjects which interest the practitioner and the surgeon. Much that was formerly theoretical in relation to this subject has now become almost absolute knowledge.

This volume includes articles from many of the most eminent men of Europe, specialists in internal medicine and in diseases of the digestive tract, such as Rosenheim, Fleiner, Leo, Strauss, Riegel, Ewald, Boas, Hirschfield, Oser, Minkowski, Stadelmann, Kraus, Neusser, Vierordt, Strasburger, Hoppe-Seyler and Nothnagel.

Subjects are treated very fully and at the same time in a concise and practical manner. Modern methods of examination, including physical and chemical measures, are clearly set forth. The diagnosis of the various diseases is fully discussed, and the treatment, including the dietary, is satisfactorily full and complete.

---

TRAITE DES MALADIES DU NEZ. Par le Docteur A. Menier. Ex-interne des Hospitaux de Paris, etc. Introduction de M. le Professeur S. Duplay. Preface de le Docteur A. Castex, Avec 178 Figures. Price 13 fr. Paris: A. Maloine, Editeur.

Dr. Menier begins his work by a description of the technique of rhinoscopic examinations with an explanation of the use of electric currents, etc.

Part two takes up the general therapeutic methods. Deformities and plastic operations of the nose follow in part three, along with malformations of the intranasal parts. Fractures, epistaxis, the various rhinites, nasal syphilis and tuberculosis are then taken up and discussed in a clear, concise manner.

The photographs of the patients before and after treatment with phototherapy, which is thoroughly described, show that the author has obtained excellent results from this method of treating lupus.

We commend Dr. Menier's book to those who wish to become familiar with the methods of treating diseases of the nose used by one of our French confreres and who desire a complete modern treatise upon the subject.

---

SOBOTTA AND MCMURRICH'S HUMAN ANATOMY. Three quarto volumes, each containing 250 pages of text and over 300 illustrations, mostly in colors. By Prof. J. Sobotta, of Würzburg. Edited, with additions, by J. Playfair McMurrich, A.M., Ph.D., Professor of Anatomy, University of Michigan. Per volume, cloth, $6.00 net. Philadelphia and London: W. B. Saunders Company.

This entirely new work on human anatomy is undoubtedly the most beautiful and practical ever produced, the numerous large quarto-size plates representing in colors the anatomic structures with a wonderful accuracy and clearness. Professor Sobotta's concise, yet clear style, so admirably maintained in the English translation, makes the work an ideal text-book for the student and an invaluable aid to the physician, surgeon and anatomist. Volume I treats of bones, ligaments, joints and muscles; Volume II of the viscera, including the heart; Volume III of the nervous and circulatory systems and the organs of the senses.

---

ABDOMINAL OPERATIONS. By B. G. A. Moynahan, M.D., (London) F.R.C.S. Leeds, England: W. B. Saunders Company, Publishers, 1906. Price .

The second edition followed the appearance of the first within a few months. The work does not include gynecological operations, nor is the surgery of the bladder and kidneys, together with hernia, included.

The work is especially full in its descriptive details of the various operations described; especially does this apply to the operations on the stomach and intestines.

All of the commonly used operations are described, but the author frankly states his preference and gives full credit to others from whose work he has drawn, Mayo and J. B. Murphy of this country, being especially mentioned.

The work has been attractively gotten up by the publishers, the type and paper being unusually good. The book should be in the library of every one doing surgery.

---

RHYTHMOTHERAPY. A discussion of the Physiologic Basis and Therapeutic Potency of Mechano-Vital Vibration, to which is added a Dictionary of Diseases, with Suggestions as to Technique of Vibratory Therapeutics. By Samuel S. Wallain, A.M., M.D. The Ouellette Press, Chicago.

Within recent years the treatment of many diseased conditions with vibration has been very satisfactory; in fact, so satisfactory that the laity, barbers and many others who know nothing of pathology are treating all kinds of conditions with vibrators. Consequently we welcome the appearance of any work that may have a tendency to place this method of treatment upon a more scientific basis.

Dr. Wallain has evidently had experience with vibration, but gives overmuch of theory. His style is breezy, fluent and alliterative, but makes rather interesting reading.

---

THE AMERICAN ILLUSTRATED DICTIONARY. All the terms used in Medicine, Surgery, Dentistry, Pharmacy, Chemistry and kindred branches, with over 100 new tables. By W. A. Newman Dorland, M.D. Fourth Revised Edition. Octavo of 836 pages, with 293 illustrations, 119 of them in colors. Philadelphia and London: W. B. Saunders Company, 1906. Flexible Morocco, $4.50 net; thumb indexed, $5.00 net.

The new fourth edition of this handy dictionary, recently issued, contains over twenty-five hundred new terms that have appeared in recent medical literature. It is an absolutely new book, defining all the terms of modern medicine and forming an unusually full vocabulary.

Of handy size, yet practically unabridged, this new dictionary seems to be just what is needed, as is evidenced by the fact that four editions have been required to meet the demand.

SAUNDERS' POCKET MEDICAL FORMULARY. By William M. Powell, M.D., author of "Essentials of Diseases of Children"; Member of Philadelphia Pathological Society. Containing 1831 formulas from the best-known authorities. With an appendix containing Posologic Tables, Formulas and Doses for Hypodermic Medication, Poisons and their Antidotes, Diameters of the Female Pelvis and the Fetal Head, Obstetric Table, Diet-lists, Materials and Drugs used in Antiseptic Surgery, Treatment of Asphyxia from Drowning, Surgical Remembrancer, Tables of Incompatibles, Eruptive Fevers, etc., etc. Eighth Edition, Adapted to the New (1905) Pharmacopœia. Philadelphia and London: W. B. Saunders Company, 1906. In flexible Morocco, with side index, wallet and flap. $1.75 net.

This handy little volume, about the size of a visiting list, will be found convenient to carry in the pocket or the buggy for quick reference. Besides many well-selected prescriptions, it contains much valuable information in tabloid form.

---

ASHTON'S PRACTICE OF GYNECOLOGY. Octavo of 1079 pages with 1046 original illustrations. By W. Easterly Ashton, M.D., LL.D., Professor of Gynecology, Medico-Chirurgical College, Philadelphia. Cloth, $6.50 net; Sheep or Half Morocco, $7.50 net.

Three editions of Dr. Ashton's excellent work have been required in eighteen months, demonstrating that the medical profession were quick to appreciate the practical value of the work. He not only tells what should be done, but also precisely *how to do it*. The 1057 drawings show in detail the procedures and operations without obscuring their purpose by unnecessary anatomic surroundings.

---

A TEXT-BOOK OF OBSTETRICS. By Barton Cooke Hirst, M.D., Professor of Obstetrics in the University of Pennsylvania. Fifth Revised Edition. Octavo of 915 pages, with 753 illustration, 39 of them in colors. Philadelphia and London: W. B. Saunders Company, 1906. Cloth, $5.00 net; Half Morocco, $6.00 net.

Immediately upon its publication Dr. Hirst's work on Obstetrics took its place as the leading text-book on the subject. That it has maintained this lead is shown by the rapid exhaustion of each edition, the new fourth edition having recently been issued.

DIET IN HEALTH AND DISEASE. By Julius Friedenwald, M.D., Clinical Professor of Diseases of the Stomach in the College of Physicians and Surgeons, Baltimore, and John Ruhrah, M.D., Clinical Professor of Diseases of Children in the College of Physicians and Surgeons, Baltimore. Second Revised Edition. Octavo of 728 pages. Philadelphia and London: W. B. Saunders Company, 1906. Cloth, $4.00 net; Half Morocco, $5.00 net.

This practical work contains a complete account of foodstuffs, their uses and chemical composition. The feeding of infants and children, of patients before and after anesthesia and surgical operations, and the latest methods of feeding after gastro-intestinal operations are all taken up in detail.

---

OBSTETRICS FOR NURSES. By Joseph B. DeLee, M.D., Professor of Obstetrics in the Northwestern University Medical School, Chicago. Second Revised Edition. 12mo of 510 pages, fully illustrated. Philadelphia and London: W. B. Saunders Company, 1906. Cloth, $2.50 net.

In this new book Dr. DeLee presents a work of the greatest value, not alone to the nurse, but also to the practitioner, upon whom the duties of a nurse often devolve in the early years of his practice. The illustrations are nearly all original.

